# Prevalence of *Borrelia burgdorferi* Sensu Lato in *Ixodes ricinus* Ticks Collected from Kaylaka Park in Pleven, Bulgaria

**DOI:** 10.3390/microorganisms10040772

**Published:** 2022-04-03

**Authors:** Alexander Blazhev, Iskren Stanilov, Lyuba Dineva Miteva, Milena Atanasova, Svetla Blazheva, Spaska Stanilova

**Affiliations:** 1 Department of Biology, Medical University-Pleven, 1 Kliment Ohridski Str., 5800 Pleven, Bulgaria; milena.atanasova-radeva@mu-pleven.bg; 2 Department of Molecular Biology, Immunology and Medical Genetics, Medical Faculty, Trakia University, 6000 Stara Zagora, Bulgaria; iskren.stanilov@trakia-uni.bg (I.S.); lyuba.miteva@trakia-uni.bg (L.D.M.); spaska.stanilova@trakia-uni.bg (S.S.); 3 Department of Immunology, University Hospital, 5800 Pleven, Bulgaria; svetlabl@abv.bg

**Keywords:** Ixodidae, vector surveillance, tick-borne disease, spirochetes, urban park

## Abstract

We aimed to determine the presence and distribution of *Borrelia burgdorferi* sensu lato (s.l.) in *Ixodes ricinus* ticks collected from urbanized and wild areas in Kaylaka Park (Bulgaria). A total of 546 ticks were collected over three years (2017–2019). The presence of *Borrelia* in 334 of the collected *I. ricinus* was detected by dark-field microscopy (DFM) and two nested PCRs (nPCR) targeting the borrelial 5S-23S rRNA intergenic spacer and Flagellin B (FlaB) gene. DFM was performed on a total of 215 ticks, of which 86 (40%) were positive. PCR was performed on 153 of the ticks. In total, 42.5% of the 5S-23S rRNA intergenic spacer and 49% of FlaB were positive. Considering as positive any single tick in which *Borrelia* sp. was detected regardless of the used method, the infection rate reached 37% (10/27) in the nymphs and 48.5% (149/307) in the adults (48.7% (77/158) females, 48.3% (72/149) males). The incidence of *B. burgdorferi* infection in *I. ricinus* did not differ statistically significantly between female, male, and nymph. This study provides evidence that Lyme disease spirochetes are present in various regions of Kaylaka Park with extremely high prevalence in their vectors.

## 1. Introduction

Hard ticks (Ixodidae) are known to transmit a wide variety of pathogenic agents with medical and veterinary importance. In Europe, an increase in both the abundance of ticks and the number of tick-borne disease cases has been reported in many countries during the past few decades [1,2]. The most prevalent tick-borne infection of humans north of the equator is Lyme disease or Lyme borreliosis (LB), whose incidence has increased in at least nine European countries over the last decade [1,2,3].

LB is a multisystemic inflammatory disease caused by infection with specific genospecies within *Borrelia burgdorferi* sensu lato (s.l.) complex and spread by Ixodid ticks [3]. It is the most common tick-borne disease of pronounced public health importance in countries with a moderate climate in the Northern Hemisphere. Its increasing incidence may be a consequence of a range of environmental factors together with changing human behavior [4]. Approximately 85,000 cases are reported annually in Europe [5,6].

The disease is caused by several genospecies of *Borrelia burgdorferi* s.l. complex. Several different genospecies of *B. burgdorferi* s.l. can be found in Europe [5,7]. The most commonly reported species in Europe are *B. afzelii*, *B. garinii*, *B. valaisiana*, and *B. burgdorferi* sensu stricto, which have also been confirmed in Bulgaria [8,9].

In Europe, the principal vector of *Borrelia* is the hard tick *Ixodes ricinus*. This hard tick is associated with deciduous and mixed forests, but during recent decades, an increase in the population of this tick and an expansion of its habitat range have been observed [7,10]. Throughout Europe, *I. ricinus* typically accounts for 90–100% of all ticks removed from humans [10]. The increase in tick densities, their increasing occurrence in urban areas, and the prolonged period of activity of these arachnids are the result of changes that have occurred in the environment, e.g., in agricultural land use, forest management, changes in the abundance and distribution of free-living animals, and climate change [11]. The observed phenomena are directly reflected in an increase in the risk of transmission of tick-borne diseases, which can be a serious problem for humans. The risk to humans of infection with *Borrelia* depends on outdoor recreational activity, on the density of tick populations, and on the infection of the ticks with *Borrelia* [12].

Areas such as parks, gardens, cemeteries, suburban leisure-time places, and playgrounds provide conditions for humans and pets to encounter potentially infected questing ticks [13,14,15,16,17,18,19,20]. Reports on tick populations in urban parks and gardens show the presence of viable tick populations as well as the presence of *Borrelia* and other tick-borne pathogens in these areas [14,16,17,18,20,21]. Studies on tick populations in Bulgaria regarding the presence of borrelia have not been conducted for many years, the data are limited and mainly for the area around Sofia [9,22].

The Kaylaka Park covers 1000 hectares and is located in the karst canyon of the Touchenitsa river. It is a local leisure hotspot, with large numbers of daily visitors and attractions such as cafes, a small zoo, an open-air theatre, sports fields, and playgrounds located about 2.5 km from the entrance. All these potentially epidemiologically highly relevant characteristics of the area make it reasonable to explore tick density and tick-borne pathogen occurrence [7,10,16,18]. The municipality is responsible for maintaining only grassplots in the part closer to the residential areas. The remainder of the park is a protected area, only spots close to large infrastructure sites are taken care of, and human intervention is minimal [23].

Reviewing the data on registered cases in the Regional Health Inspectorate-Pleven, it is found that over the last 12 years there has been an increasing incidence of LB [24]. Despite the growing number of registered cases of LB in the Pleven region, surveys for potential sources of infection have not been conducted. Thus, we have conducted a series of studies in order to reveal the potential sources of human infection. In a previous survey, we found persistent tick populations in the park area, mainly of the species *Ixodes ricinus* [23].

To date, no study on the presence of pathogens in ticks has been carried out in Kaylaka Park and Pleven District. This study aimed to assess the prevalence of *Borrelia burgdorferi* s.l. by dark-field microscopy (DFM) and/or nested polymerase chain reaction (nPCR) in questing ticks from different areas of the Kaylaka Park.

## 2. Materials and Methods

### 2.1. Study Sites

**Figure 1:**
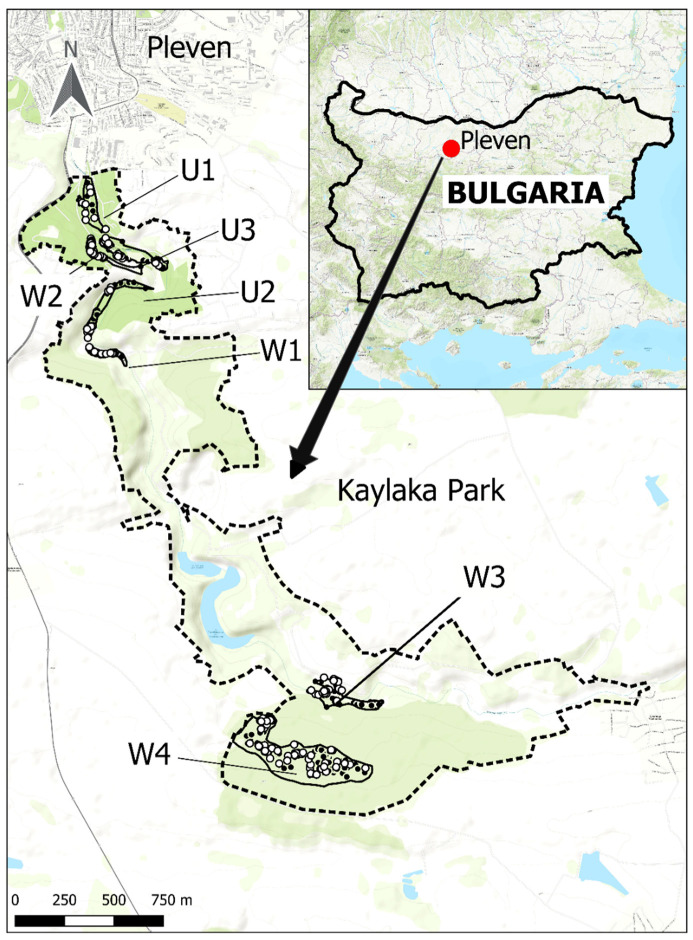
Location of the Kaylaka Park and sampling sites urban (U1–U3) and wild (W1–W4) in Bulgaria. The boundaries of the Kaylaka Park protected area are represented by a dotted line. All sample collection areas are represented by a solid line. Each collected tick is marked with a dark circle. Ticks that are positive for *Borrelia* are represented by a white circle.

Seven sampling sites in Kaylaka Park were investigated over a period of three years (2017–2019). The sites in the park were divided into two categories, urban and wild areas (Figure 1), depending on the daily presence of people and the transformation of the area. The urban area included three sites located near the city of Pleven (U1–U3). Sampling sites U1 to U3 included a central alley of about 2.8 km. Along the alley are deciduous and coniferous trees and grass spots. The urban sites are exposed to strong anthropogenic influence, highly frequented by visitors, maintained by gardening (e.g., mowing), and intensively used by citizens for different outdoor activities, especially regularly walking dogs, walking with children, cycling, jogging, etc. Urban areas also have a high degree of human-induced landscape transformation, including highly transformed areas of urban infrastructure, residential areas, and alleys, including playgrounds, a zoo, swimming pools, cafes, restaurants, sports paths, etc. Rodents and birds, as well as synanthropic carnivores such as dogs (*Canis lupus familiaris*), hedgehogs (*Erinaceus europaeus*), and European polecat (*Mustela putorius*), inhabit all the urban sites in the park [25].

The second type of site was natural (wild) and included four areas in the park further from the city (W1–W4). The second category included semi-natural woodland areas with natural and managed forests and low-transformed settlement foci, more rarely visited by people. It is known that they are permanently inhabited by large mammals, including a free-living population of roe deer (*Capreolus capreolus*), wild boar (*Sus scrofa*), foxes (*Vulpes vulpes*), European badger (*Meles meles*), and jackals (*Canis aureus*). Large numbers of rodents, insectivores, birds, and reptiles are also common (personal observation) [25]. These areas are mainly visited by forestry workers, tourists, collectors of herbs, or mushroom pickers during specific periods of the year. Information on the sampling areas has been described in a previous study [23].

### 2.2. Tick Collection

Campaigns for tick sampling were performed from March to June for three years (2017 and 2019) using the flagging method with a white flannel flag (contact surface 1 m^2^) attached to a handle. The exact area flagged during each sampling depended on the available time and weather conditions and on local tick density (generally less flagged in high-density spots). Different flagging spots in sampling sites were selected separately for each session. The choice was based on the operator’s assessment of suitable tick microhabitats and covered varied areas and biotopes within the sampling sites. The days of sampling were determined based on the following criteria: rainless, wind speed of less than 3 according to the Beaufort wind force scale, air temperature above 12 °C. The collected ticks were placed in individual 1.5 mL safe-lock Eppendorf tubes (Eppendorf AG, Hamburg, Germany) and were transferred to the laboratory where they were washed with sterile water in an ultrasonic cleaner (Silver Crest, HOYER Handel GmbH, Hamburg, Germany). They were then identified to the species level, developmental stage, and sex according to the morphological keys provided in Georgieva [26] and Estrada-Peña et al. [27] using a stereo microscope (Olympus SZ4045, Olympus American Inc., Melville, NY, USA).

### 2.3. Geographic Integration

Geographic mapping was conducted on Quantum Geographic Information Systems (QGIS version 3.18.0, QGIS Development Team, GNU General Public License, Essen, Germany) with the World Geodetic System 1984 (WGS 84) standard of coordinate referencing. All geographical data and linked population data were imported and analyzed using Microsoft Excel (version 14.0; Microsoft, Redmond, WA, USA).

### 2.4. DNA Extraction and PCR

Genomic DNA was extracted from individual ticks with the NucleoSpin Tissue Mini Kit for DNA from cells and tissue (MACHEREY-NAGEL GmbH & Co. KG, Düren, Germany). The extracted DNA was stored at −70 °C until PCR analysis.

### 2.5. Borrelia burgdorferi s.l. DNA Detection by Polymerase Chain Reaction (PCR)

To detect the presence of the Borrelia’s DNA, we performed two nested PCRs (nPCR) targeting (i) a non-coding region, the 5S-23S rRNA intergenic spacer (PCR_5S-23Sigs_), and (ii) a protein-coding gene for Flagellin B (FlaB). The sequences of used primers are shown in Table 1.

For the initial PCR run, we used the external primers in combination with 3 µL template DNA, and for nested PCR we used 2 µL from the first PCR reaction product. The reaction setup was performed in 20 µL final volume containing 2 µL 10× PCR buffer, 1.5 mM MgCl_2_, 10 mM dNTP, and 1 U Taq DNA polymerase. The amplification program was as follows: 95 °C for 5 min; 35 cycles of 95 °C for 30 s, 55 °C for 30 s, and 40 s at 72 °C, and a final extension at 72 °C for 5 min. The second round of PCR was performed similarly to the first reaction, except for the use of inner primers, 2 μL product of the first PCR, and an annealing temperature at 59 °C.

For the second nested PCR (nPCR*_FlaB_*), which identified the presence of *FlaB* gene sequence in *Borrelia burgdorferi* s.l., we used primers presented in Table 1 and the PCR setup as described by Wills et al., 2018 [31].

As a positive control, we used the purified genomic DNA (isolated from *Borrelia burgdorferi*; strain B31, [ATCC^®^35210D5™], delivered by LGC, Germany, Hanover) diluted to a concentration of 10 ng/μL for PCR amplification.

### 2.6. Agarose Gel Electrophoresis and Imaging

All PCR products were analyzed on a 1.5% agarose gel electrophoresis in TAE buffer (40 mM Tris-acetate, 2 mM EDTA (pH 8.5)) at 5 V/cm for 30 min, stained with ethidium bromide. DNA Ladder (100 bp) was applied for an estimation of the obtained product size. The results of the PCR amplification were visualized using Herolab gel documentation system and its analysis software E.A.S.Y (Herolab GmbH Laborgeräte, Wiesloch, Germany). DNA extraction, fragment amplification, and agarose gel electrophoresis were performed in separate rooms.

### 2.7. Dark-Field Microscopy (DFM)

A part of the tick midgut contents was placed in a drop of phosphate buffered saline (PBS) solution (8.5 g of NaCl, 0.9 g of Na_2_HPO_4_, and 0.2 g of KH_2_PO_4_ per liter, pH 7.2). Then, it was immediately covered with a thin coverslip, and the slide was examined by DFM microscopy (LEICA DM500, darkfield slider, Leica Microsystems Heerbrugg, Switzerland) for the presence of live spirochetes by viewing 100 fields at a magnification of 400×. Typical movement around the longitudinal axis, morphology, and size were used as the identification criteria for the *Borrelia* sp. The remaining portion of the tick was stored in 70% ethanol at 4 °C for PCR analysis.

### 2.8. Statistical Analysis

Statistical analyses were performed using SPSS 23.0 (SPSS, Inc., Chicago, IL, USA) and Microsoft Excel (2007) for Windows.

Differences in tick infection prevalence between sexes, years, and localities and depending on the detection technique applied were statistically analyzed by means of a nonparametric Chi-square test. A test reliability analysis using the kappa statistic was performed to determine consistency among DFM microscopy and nPCR tests. The data were presented as kappa coefficient (k) and 95% confidential interval (95% CI). In any case, the differences were considered significant with *p* values of less than 0.05.

## 3. Results

During the study period (2017–2019), a total of 546 ticks were collected, out of which 253 (46.3%) were females, 238 (43.6%) were males, 54 (9.9%) were nymphs, and only one was larva (0.2%). Details of ticks collected by year, sex, and stage of development are presented in Table (Table 2).

**Figure 2:**
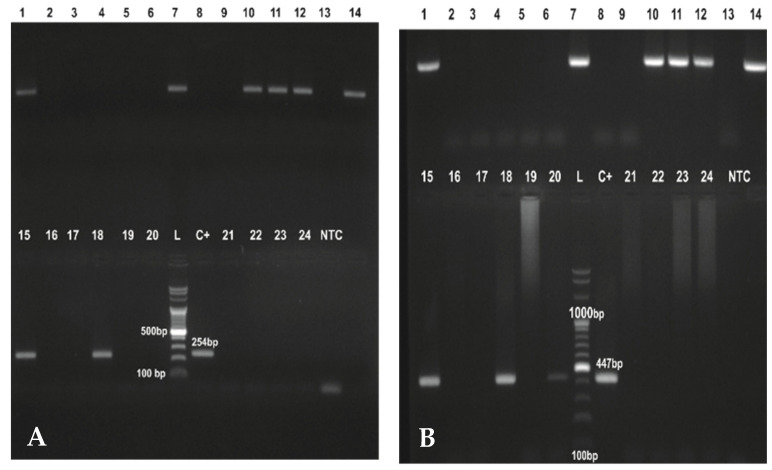
PCR products of nPCR of *Borrelia burgdorferi* s.l. *5S-23S intergenic spacer* (panel (**A**)) and *FlaB* (panel (**B**)) analyzed on a 1.5% agarose gel electrophoresis. The same DNA templates were used in both nPCRs. L: DNA ladder in 100 bp increments; C+: DNA from positive control; NTC: non-template control.

A total of 334 ticks were examined for the presence of *Borrelia burgdorferi* s.l. Dark-field microscopy was performed on a total of 215 ticks, with 86 (40%) positive for *Borrelia* sp. Polymerase chain reaction was performed on 153 of the collected ticks. The PCR results for *5S-23S* intergenic spacer and *FlaB* gene identification are presented in Figure 2.

From the nPCR*_5S-23Sigs_* testing, 65 (42.57%) were positive. The PCR results for the flagellin gene showed 75 (49%) to be positive. Detailed results of the survey of *Ixodes ricinus* infectivity with *Borrelia* by area within Kaylaka Park and by years of collection are presented in Table 3 and Table 4, respectively.

The results reported were obtained from independent/non-selective testing of the collected ticks. One hundred and fifty-three ticks were tested for the presence of *B. burgdorferi* s.l. using nPCR*_FlaB_* and nPCR*_5S-23Sigs_*. DFM microscopy was performed on 215 ticks, of which 35 were included in both PCRs. From the agreement (kappa statistics) between the results of DFM microscopy, nPCR*_5S-23Sigs_*, and nPCR*_FlaB_* analysis, the kappa values for all three comparisons indicated different level of agreement. Comparing all ticks tested with PCR, the agreement between nPCR*_FlaB_* and nPCR*_5S-23Sigs_* was substantial; kappa = 0.764 (*p* < 0.001), 95% CI (0.662 to 0.866). A substantial agreement was shown by comparison between the two nPCRs in the sample of 35 ticks tested simultaneously with all three methods; κ = 0.770 (*p* < 0.001), 95% CI (0.560 to 0.981). The kappa values for comparisons of DFM microscopy and nPCR*_5S-23Sigs_* and DFM and nPCR*_FlaB_*, were κ = 0.422 (*p* < 0.008), 95% CI (0.139 to 0.706) (moderate agreement) and κ = 0.397 (*p* < 0.016), 95% CI (0.094 to 0.700) (fair agreement), respectively.

The positive and negative rates in all tests of the examined ticks were 16 and 7, respectively, which was collectively 65.7% of the samples. Seven of the ticks (20%) in which *Borrelia* was detected by DFM were not detectable by either nPCR*_5S-23Sigs_* or nPCR*_FlaB_*. One positive and three negative on DFM yielded a positive result by nPCR*_FlaB_*, and two negatives on DFM yielded a positive by nPCR*_5S-23Sigs_*. Three of the samples that tested negative with nPCR*_5S-23Sigs_* were reported as positive when tested with nPCR*_FlaB_*.

As different used methods for the detection of *Borrelia burgdorferi* s.l. have several advantages and disadvantages, we considered ticks as positive when *Borrelia* was detected regardless of the method of testing (DMF and/or PCRs positive tick). Irrespective of the insignificant differences in the rate of the infected *Borrelia burgdorferi* s.l. between 2017–2019 years (*χ*^2^ = 3.955; *df* = 2; *p* = 0.138), it should be noted that, over the years, the percentage of positive ticks slightly increase. In 2019, 52.5% positive ticks were detected, while in 2017, just 40.9% were detected (*χ*^2^ = 3.395; *p* = 0.065).

**Figure 3:**
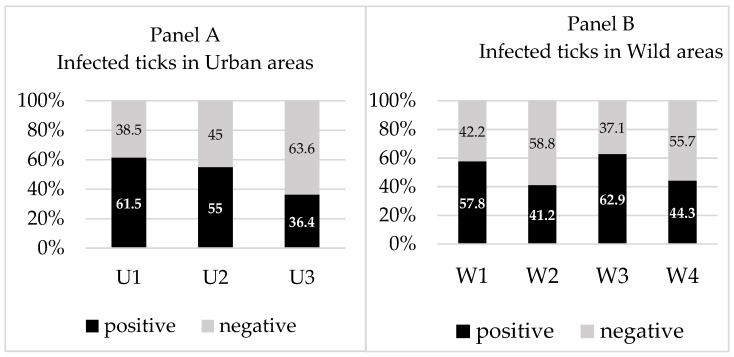
The rate of DMF and/or PCR positive ticks in which *Borrelia* sp. was detected regardless of the method of testing in urban (U1–U3) and wild (W1–W4) areas within Kaylaka Park. Data are presented as percentages.

There was no statistically significant difference in prevalence of *Borrelia* sp. between the ticks sampled in urban and wild areas regardless of the method of testing (44.3% vs. 48.8%; *χ*^2^ = 0.517; *p* = 0.472). Among urban areas (Figure 3, Panel A), there was a tendency for a lower incidence of infected ticks in area U3 (36.4%) compared to other urban areas, with no statistical significance (*χ*^2^ = 3.761; *p* = 0.052). Within wild areas (Figure 3, Panel B), the lowest incidence of infected ticks was observed in W2 (41.2%), followed by W4 (44.3%), W1 (57.8%), and W3 (62.9%).

Considering as positive any single tick in which *Borrelia* sp. was detected by DFM and/or PCR (any method), the infection rate reached 48.5% (*n* = 149/307) in adult ticks. Borrelia were detected in 48.7% (*n* = 77/158) of the females, 48.3% (*n* = 72/149) of the males, and 37.0% (*n* = 10/27) of the nymphs. The prevalence of *Borrelia* sp. infection (by any method) in *I. ricinus* did not differ statistically significantly between females, males, and nymphs (*χ*^2^ = 1.320; *df* = 2; *p* = 0.517).

## 4. Discussion

Recent decades have seen an increase in outdoor recreational activities. Kaylaka Park provides numerous outdoor attractions for the citizens of Pleven. On the other hand, most of the park is a protected area, located in the canyon of the Tuchenica River, which has favorable conditions for the populations of various free-living animals. In the area of the park, there are stable populations of large and small mammals, numerous species of birds (migratory and resident), and reptiles. All these features ensure ideal conditions for maintaining the full life cycle of *I. ricinus* and the pathogens it spreads. Many studies have demonstrated the presence of *Borrelia burgdorferi* s.l. in ticks collected from urban and peri-urban environments [10,14,17,32].

For Bulgaria, a limited number of studies on the prevalence of *B. burgdorferi* s.l. in *Ixodes ricinus* have been carried out, and the more recent published data concern the Sofia area for the period 2000–2001 [9,22]. In these papers, Christova et al. investigated 113 adult ticks collected in 2000 and 72 collected in 2001, where overall Borrelia prevalence was 41% and 31%, respectively. Similar surveys of *I. ricinus* for the presence of *B. burgdorferi* s.l. in other countries of the Balkan Peninsula yielded large differences in the prevalence of ticks. In a study of ticks (*n* = 30) from Bosnia and Herzegovina, no bacteria of the *B. burgdorferi* s.l. were detected [33]. A relatively low prevalence in ticks was shown by studies from Romania; the prevalence of Borrelia was between 0.75% and 18.8% [34,35]. In the European part of Turkey, infection rates were 38.7% in Istanbul and 11.4% in the Kirklareli area [36]. A high prevalence of tick infectivity was reported in studies from Serbia. Milutinović et al., in a study of 18 different areas in Serbia, found 42.5% [37], while in another survey from Serbia, 49% of collected ticks were positive for *B. burgdorferi* s.l. [19].

Rauter and Hartung, in their meta-analysis, classify areas according to tick infection rates, with low infection rates (nymphs ≤ 11%; adults ≤ 20%), high infection rates (nymphs > 11%; adults > 20%), and extremely high (>30%). Their analysis showed that the average infection rate was 13.7% out of a total of 112, 579 ticks tested in Europe over the period from 1984 to 2003 [12]. The results of this study identified Kaylaka Park as an extremely high infection area. Another meta-analysis based on the detection of *Borrelia burgdorferi* s.l. mainly by PCR (2010–2016) showed that the overall mean prevalence of *B. burgdorferi* s.l. in *I. ricinus* ticks reached 12.3% in Europe [38].

Despite the increasing number of LB cases both in Europe and in Pleven [2,13,39], the tick populations in Kaylaka Park had not yet been examined for infection with *Borrelia burgdorferi* s.l. In this study, we confirmed the presence of *Borrelia* spirochetes by DFM, and of *B. burgdorferi* s.l. specifically, by nPCR in both maintained urban areas and wild areas of the park. There was no statistically significant difference in tick infectivity between urban and wild areas. However, U3 was noted to have the lowest infection rate with a relatively large number of examined ticks. The flagging area in this zone was the most visited by citizens due to the various attractions in the vicinity; accordingly, the maintenance of the green areas is constant. In the other two urban areas, the high infection rates are probably due to the lower number of ticks tested. Studies with a higher number of *I. ricinus* examined are required in those areas to acquire more robust results. In the wild areas, W3 and W1 sampling sites had the highest infection rates. Both areas are located at the bottom of the Tuchenica River canyon where there is dense deciduous vegetation, shrubs, and herbaceous plants. This contributes to the diversity of different mammals, birds, and reptiles that are natural hosts of *I. ricinus*. Moreover, the moisture from the river helps the survival of tick populations and maintains the enzootic cycle of the *B. burgdorferi* s.l. [40].

The agreement between both PCRs was substantial, while the agreement between nPCR*_5S-23Sigs_* and nPCR*_FlaB_* with DFM was moderate and fair, respectively. One reason for the discrepancy in the detection of Borrelia by PCRs and DFM can be explained by the small number of spirochetes in a tick. In an attempt to overcome this possibility, nested PCR was chosen in our study. Nested PCR was designed to improve the sensitivity and specificity of DNA templates in low abundance. In addition, we chose two target genes, one non-protein-coding and one encoding for Flagellin B, to increase the specificity of the method. Both target genes are widely used in molecular biological detection and identification of *Borrelia burgdorferi* s.l. In addition, it should be noted that DFM, used as a standard procedure in the past, does not allow for distinguishing between different species of the genus *Borrelia*. Furthermore, if not used correctly, they may lead to an underestimation of Borrelia infection rates [41]. On the other hand, the prevalence of LB spirochetes in *I. ricinus* ticks reported in the past could also be overestimated due to the misidentification of the relapsing fever spirochete *B. miyamotoi* by microscopy [42].

Notwithstanding the foregoing, PCR methods are extremely precise and are finding increasing applications in various fields of biology and medicine. DFM microscopy is an easy, inexpensive, and accessible method suitable for pilot studies on the distribution of borrelia in ticks. Despite the lack of complete concordance of the results, used together or separately they provide a good picture of the distribution of *B. burgdorferi* s.l. among *I. ricinus* populations.

However, it should be noted that borrelia-infected ticks were found in all areas that were surveyed, suggesting that people using such habitats may come into contact with infected ticks. We demonstrated the presence of *B. burgdorferi* s.l. in ticks inhabiting Kaylaka Park. They are present in both wild unmaintained areas and urbanized areas of the park. Visitors should take measures to protect themselves from ticks and follow treated paths and walkways. Physicians should be aware that patients may encounter a tick infected with *B. burgdorferi* s.l. in the park and may subsequently become infected with LD.

The high incidence of Borrelia-infected ticks potentially exposes people using this area for recreation to the risk of infection [43]. Further research on the reporting of tick bite cases among visitors of Kaylaka Park may be useful here. In such high-risk locations, regular maintenance of lawns through treatment against blood-feeding arthropods as well as the posting of information signs about the presence of ticks may be useful in reducing human exposure to ticks. Collecting data on tick populations and the prevalence of various tick-borne pathogens in these types of suburban parks can help to focus public health campaigns [14], especially if visitors in such areas have limited knowledge of ticks and pathogens.

A limitation of this study is the non-identification of the different genospecies of the *B. burgdorferi* s.l. complex. Different species of the complex have different clinical manifestations, and a logical sequel of this study would be to determine the species found in the Kaylaka Park area and their relationship to symptomatology among LB patients from the area.

## 5. Conclusions

Our survey reports the presence of *Borrelia burgdorferi* sensu lato in questing *Ixodes ricinus* collected from Kaylaka Park, Pleven, Bulgaria. The data identify Kaylaka Park as an extremely high infection area, with no significant differences between investigated urban and wild areas in the follow-up period.

## Figures and Tables

**Table 1:** Inner and outer primers for nPCR of *Borrelia burgdorferi* s.l. FlaB and 5S-23S intragenic spacer.

Primer Name	Gene	Target Sequence (5′-3′)	Amplicon Size	Annealing Temperature	References
Primer 23S3 Out	*rrf (5S)-rrl (23S) intergenic spacer*	CGACCTTCTTCGCCTTAAAGC	411 bp	55 °C	Chu et al., 2008 [28] Schwartz et al., 1992 [29]
Primer 23Sa Out		TAAGCTGACTAATACTAATTA CCC			
Primer 1 In	*rrf (5S)-rrl (23S) intergenic spacer*	CTG CGA GTT CGC GGG AGA	254 bp	59 °C	Postic et al., 1994 [30]
Primer 2 In		TCC TAG GCA TTC ACC ATA			
FlaB Out Fw	*FlaB*	GCATCACTTTCAGGGTCTCA	503 bp	55 °C	Wills et al. [31]
FlaB Out Rv		TGGGGAACTTGATTAGCCTG			
FlaB In Fw		CTTTAAGAGTTCATGTTGGAG	447 bp	58 °C	
FlaB In Rv		TCATTGCCATTGCAGATTGT			

**Table 2:** Collection data of *Ixodes ricinus* ticks by years and stage/gender.

Year	Stage_Gender				Total
	Male	Female	Nymph	Larva	
2017	58	99	12	0	169
2018	71	51	10	1	133
2019	109	103	32	0	244
Total	238	253	54	1	546

**Table 3:** Results of *Ixodes ricinus* testing for the presence of *Borrelia burgdorferi* s.l. within the Kaylaka Park study areas.

Area	Type of Test				Detection of *Borrelia* sp. in *I. ricinus* by Any Test Percentage of Tick Infection (%) and Number of Positive Samples (*n*)
	Number of Collected Ticks	PCR 23S/5S	PCR FlaB	DFM	
		Tick Infection (%) and Number of Positive to Tested Samples (*n*)	Percentage of Tick Infection (%) and Number of Positive to Tested Samples (*n*)	Percentage of Tick Infection (%) and Number of Positive to Tested Samples (*n*)	
**Urban**	**192**	**35.7 (*n* = 15/42)**	**47.6 (*n* = 20/42)**	**42 (*n* = 22/52)**	**44.3 (*n* = 39)**
U1	40	45.5 (*n* = 5/11)	54.5 (*n* = 6/11)	100 (*n* = 2/2)	61.5 (*n* = 8)
U2	50	0 (*n* = 0/3)	33.3 (*n* = 1/3)	58.8 (*n* = 10/17)	55.0 (*n* = 11)
U3	102	35.7 (*n* = 10/28)	46.4 (*n* = 13/28)	30.3 (*n* = 10/33)	36.4 (*n* = 20)
**Wild**	**354**	**45 (*n* = 50/111)**	**49.5 (*n* = 55/111)**	**37.4 (*n* = 64/163)**	**48.8 (*n* = 120)**
W1	79	53.7 (*n* = 22/41)	53.7 (*n* = 22/41)	25 (*n* = 1/4)	57.8 (*n* = 26)
W2	58	16.7 (*n* = 2/12)	33.3 (*n* = 4/12)	40.9 (*n* = 18/44)	41.2 (*n* = 21)
W3	83	52.9 (*n* = 9/17)	58.8 (*n* = 10/17)	66.7 (*n* = 12/18)	62.9 (*n* = 22)
W4	134	41.5 (*n* = 17/41)	46.3 (*n* = 19/41)	34 (*n* = 33/97)	44.3 (*n* = 51)
**Total**	**546**	**42.5 (*n* = 65/153)**	**49 (*n* = 75/153)**	**40 (*n* = 86/218)**	**47.6 (*n* = 159)**

**Table 4:** Results from testing by DFM, nPCR*_5S-23Sigs_,* and nPCR*_FlaB_* for Borrelia infection in *I. ricinus* during the collecting campaign (2017–2019).

Year of Collection	Total	Method	Tested Ticks	Results	
	Collected Ticks			Positive	Negative
2017	169	DFM	124	49 (39.5%)	75 (60.5%)
		nPCR*_5S-23Sigs_*	16	5 (31.3%)	11 (68.8%)
		nPCR*_FlaB_*	16	8 (50.0%)	8 (50.0%)
2018	133	DFM	77	33 (42.9%)	44 (57.1%)
		nPCR*_5S-23Sigs_*	28	8 (28.6%)	20 (71.4%)
		nPCR*_FlaB_*	28	10 (35.7%)	18 (64.3%)
2019	244	DFM	14	4 (28.6%)	10 (71.4%)
		nPCR*_5S-23Sigs_*	109	52 (47.7%)	57 (52.3%)
		nPCR*_FlaB_*	109	57 (52.3%)	52 (47.7%)

## Data Availability

Not applicable.
